# Concerns regarding the validity of nutrition self-efficacy questionnaire among Iranian elderly population

**DOI:** 10.1186/s41043-022-00282-1

**Published:** 2022-02-17

**Authors:** Saeed Pahlevan Sharif, Navaz Naghavi, Hamid Sharif Nia

**Affiliations:** 1 Taylor’s University, Lakeside Campus, Subang Jaya, Malaysia; 2 Global Centre for Modern Ageing, Adelaide, South Australia 5042 Australia; 3 Amol Faculty of Nursing and Midwifery, Mazandaran University of Medical Sciences, Sari, Iran

**Keywords:** Exploratory factor analysis, Construct validity, Reliability, Psychometric

## Abstract

There are some statistical concerns regarding a recently published article which has claimed to develop and psychometrically evaluate an instrument to assess the nutrition self-efficacy among Iranian elderly population.

Dear Editor,

We read with interest the article titled “Nutrition self-efficacy assessment: designing and psychometric evaluation in a community-dwelling elderly population” by Shamsalinia, Ghadimi [1] published in the *Journal of Health, Population and Nutrition* in 2019. Using mixed methods, the authors developed and psychometrically evaluated an instrument to assess the nutrition self-efficacy among Iranian elderly population. However, there are some serious concerns about the reported results that we intend to share with the editor.

Authors stated that “an EFA using principal components analysis was undertaken to explore the underlying structure of the NSEQ” ([1], P. 5). However, exploratory factor analysis (EFA) and principal component analysis (PCA) are two different methods for different purposes [2, 3]. Although in some studies EFA and PCA incorrectly have been used interchangeably (See [3]), as Fokkema and Greiff [4] stated “PCA should never be referred to as (exploratory) factor analysis” (p. 401). Indeed, PCA is more suitable for reducing observed variables into smaller groups of components rather than exploratory extracting underlying factors (latent constructs) [3].

Also, to assess construct reliability, convergent validity and discriminant validity of the instrument, authors claimed to compute composite reliability (CR), average variance extracted (AVE), maximum shared variance (MSV), and average shared variance (ASV) using the results obtained from PCA. While conducting PCA or even EFA to compute CR, AVE, and MSV is questionable, the computed values for the measures are not consistent with the factor loadings reported in the paper [5]. We prepared Table 1 in which CR and AVE values reported in the paper are presented in correspondence with our computed values using the reported factor loadings of the three constructs following the formulas below (See [5–7]).

$${\text{AVE}} = \frac{{\mathop \sum \nolimits_{i = 1}^{n} L_{i}^{2} }}{n}$$

where *i* is the number of items ranging from 1 to *n*. *n* represents total number of items and *L*_*i*_ represents the standardized factor loading of item number *i*.

$${\text{CR}} = \frac{{\left( {\mathop \sum \nolimits_{i = 1}^{n} L_{i} } \right)^{2} }}{{\left( {\mathop \sum \nolimits_{i = 1}^{n} L_{i} } \right)^{2} + \left( {\mathop \sum \nolimits_{i = 1}^{n} e_{i} } \right)}}$$

where *i* denotes the number of items ranging from 1 to *n*. *n* represents total number of items and *L*_*i*_ represents the standardized factor loading of the item number *i*. *e*_*i*_ is the unexplained variance of item number *i* by the construct.

**Table 1:** Comparing AVE and CR reported in the paper with what we computed based on the reported factor loadings

	Reported in the paper	Computed based on the factor loadings reported in the paper		
	AVE	CR	AVE	CR
Adherence efficacy	0.577	0.901	0.260	0.829
Preventive behaviors	0.514	0.912	0.246	0.763
Information effectiveness	0.636	0.913	0.291	0.662

*CR* composite reliability, *AVE* average variance extracted

As it is shown, none of the computed AVE values meet the convergent validity threshold of AVE greater than 0.5. Moreover, contrarily to the claimed statement in the paper, CR of information effectiveness is below 0.7 deviating from the construct reliability requirement. Therefore, in contrast to the authors’ claim, this study has failed to introduce a reliable construct to measure nutrition self-efficacy among Iranian elderly population.

Besides, our concern continues further to the reported ASV and MSV. Shared variance is the square of the correlation between any two constructs. Therefore, ASV of a construct is the mean of the square of the correlation between the construct and other constructs. Also, MVS of a construct is the largest square of the correlation between the construct and other constructs [6, 8]. In the study by Shamsalinia et al. [1] there are three constructs in the measurement model and accordingly, there are three covariances between the three constructs. As three different MSV values have been reported in the results, each of the MSV values basically is one of the shared variances between two of the constructs. This means that the reported ASV value for each of the three constructs should be the mean of two of the MSV values. In other words, based on the reported MSV values, ASV values should be 0.353, 0.355, and 0.374 which are different from the results reported in the paper (i.e., 0.329, 0.349, and 0.358). There are more concerns about the reported results. For example, rather than reporting both lower bound and upper bound, only one value for the 95% confidence intervals for Cronbach’s alpha is reported. Also, it is not clear what 0.865 and 0.896 are in the Spearman rank-order correlation coefficient table.

The existence of high level of statistical errors in medical journals, intentionally or unintentionally, has been always caused much concern. Construct reliability and validity lie at the heart of competent and effectiveness of an instrument [9, 10]. Due to the salient statistical errors in assessing the reliability and validity of the construct, it remains a big concern if this construct is valid to be accessed for future research. Moreover, having it published in an open access journal, it amplifies the importance to warn the irreparable damage it may cause. Providing the collected data of the published paper to the reader might be a solution to prevent such manipulations in the future.

## Data Availability

Not applicable.

## Abbreviations

AVE: Average variance extracted
ASV: Average shared variance
CR: Composite reliability
EFA: Exploratory factor analysis
MSV: Maximum shared variance
PCA: Principal component analysis
