# Correction to: Concerns regarding the validity of nutrition self-efficacy questionnaire among Iranian elderly population

**DOI:** 10.1186/s41043-022-00290-1

**Published:** 2022-03-25

**Authors:** Saeed Pahlevan Sharif, Navaz Naghavi, Hamid Sharif Nia

**Affiliations:** 1 Taylor’s University, Lakeside Campus, Subang Jaya, Malaysia; 2 Global Centre for Modern Ageing, Adelaide, SA 5042 Australia; 3 Amol Faculty of Nursing and Midwifery, Mazandaran University of Medical Sciences, Sari, Iran

## Correction to: Journal of Health, Population and Nutrition (2022) 41:3 https://doi.org/10.1186/s41043-022-00282-1

Following publication of the original article [1], the authors reported an error in the affiliation of the author Sharif Nia. The correct affiliation of the author is indicated hereafter and the changes have been highlighted in **bold typeface**.

The incorrect affiliation of Sharif Nia reads:

^1^Taylor’s University, Lakeside Campus, Subang Jaya, Malaysia.

The correct affiliation of Sharif Nia should read:

^**3**^ **Amol Faculty of Nursing and Midwifery, Mazandaran University of Medical Sciences, Sari, Iran.**

The affiliation for Sharif Nia has been updated in this correction and the original article [1] has been corrected.
